# CMAX3: A Robust Statistical Test for Genetic Association Accounting for Covariates

**DOI:** 10.3390/genes12111723

**Published:** 2021-10-28

**Authors:** Zhongxue Chen, Yong Zang

**Affiliations:** 1 Department of Epidemiology and Biostatistics, School of Public Health, Indiana University Bloomington, Bloomington, IN 47401, USA; zc3@indiana.edu; 2 Department of Biostatistics and Health Data Science, School of Medicine, Indiana University, Indianapolis, IN 46202, USA; 3 Center for Computational Biology and Bioinformatics, Indiana University, Indianapolis, IN 46202, USA

**Keywords:** MAX3 test, genetic model, score test, risk allele, genotype, phenotype, GWAS

## Abstract

The additive genetic model as implemented in logistic regression has been widely used in genome-wide association studies (GWASs) for binary outcomes. Unfortunately, for many complex diseases, the underlying genetic models are generally unknown and a mis-specification of the genetic model can result in a substantial loss of power. To address this issue, the MAX3 test (the maximum of three separate test statistics) has been proposed as a robust test that performs plausibly regardless of the underlying genetic model. However, the original implementation of MAX3 utilizes the trend test so it cannot adjust for any covariates such as age and gender. This drawback has significantly limited the application of the MAX3 in GWASs, as covariates account for a considerable amount of variability in these disorders. In this paper, we extended the MAX3 and proposed the CMAX3 (covariate-adjusted MAX3) based on logistic regression. The proposed test yielded a similar robust efficiency as the original MAX3 while easily adjusting for any covariate based on the likelihood framework. The asymptotic formula to calculate the *p*-value of the proposed test was also developed in this paper. The simulation results showed that the proposed test performed desirably under both the null and alternative hypotheses. For the purpose of illustration, we applied the proposed test to re-analyze a case-control GWAS dataset from the Collaborative Studies on Genetics of Alcoholism (COGA). The R code to implement the proposed test is also introduced in this paper and is available for free download.

## 1. Introduction

The goal of a genetic association study is to identify candidate genetic factors that are associated with specific disease status [1,2,3,4,5,6]. Toward this goal, a variety of statistical methods have been developed to test the association between disease and genetic variants [7,8,9,10,11,12]. Among all the available methods, logistic regression is arguably the most popular one for a binary disease outcome (e.g., case-control study) because it accounts for other covariate effects when it tries to detect the genetic effect. In the logistic regression, the logit transformation of the disease risk is linked with a linear combination of genetic effects and covariate effects. Hence, this method naturally controls the covariate effects by estimating the corresponding coefficients from the regression term [6]. 

To use the logistic regression to test for genetic association, we need to specify the null and alternative hypotheses, with the null representing the absence of genetic effect and the alternative representing the presence of genetic effect. When a diallelic marker is used for a genetic association study, under the null hypothesis, an individual’s risk (e.g., the probability of having the disease given the genotype) of having the disease should be the same regardless of their genotype at the marker. Under the alternative, the penetrance should increase with the number of risk alleles in the genotype. In other words, the penetrance increases with the number of disease alleles under the alternative hypothesis. 

Three genetic models, named recessive (REC), additive (ADD), and dominant (DOM), are commonly used to sort the penetrance under the alternative hypothesis. Specifically, the penetrance of having one risk allele is equal to the penetrance of having no risk alleles, two risk alleles, and the middle of these two under the REC, DOM, and ADD models, respectively. If the underlying genetic model can be pre-specified, then an optimal set of scores can be used in the regression model representing the underlying genetic model, which can maximize the power of the statistical test. Unfortunately, for many complex diseases, the genetic model is typically unknown, and a mis-specified genetic model can result in a substantial loss of statistical power [13]. 

When the genetic model cannot be pre-determined, an ADD model is often times considered as a default model for GWAS given its relative robustness for identifying dominant and additive effects [14]. The likelihood ratio test (LRT) can also be used as a robust statistical test, which uses two dummy variables to represent the genetic effect [15]. Under the null hypothesis, the LRT asymptotically follows a chi-square distribution with two degrees of freedom, based on which the *p*-value can be easily calculated. However, the LRT sacrifices the efficiency of the test because it completely ignores the restricted penetrance space under the alternative hypothesis. On the other hand, the MAX3 test, which takes the maximum absolute value for the three test statistics under the REC, ADD, and DOM models, has been proposed as a trade-off statistic balancing the power and robustness of the statistical test against genetic model uncertainty [16,17,18,19]. The MAX3 test retains the flexibility of the underlying genetic model but also restricts the alternative space to three realistic situations rather than the whole space. Therefore, it obtains desirable statistical power regardless of which one is the true underlying genetic model. However, due to the multiple comparison issue, the asymptotic distribution of the MAX3 test statistic does not follow a standard normal distribution even under the null hypothesis, and the *p*-value needs to be calculated accordingly. 

The MAX3 is arguably one of the most popular robust tests and has been applied in many genetic association studies. For example, Zhang et al. [20] has applied the MAX3 test to explore the association of *HOX* transcript antisense intergenic RNA SNPs with the risk of hepatocellular carcinoma in a Southern Chinese population. Ren et al. [21] used the MAX3 test to evaluate the association between a disintegrin and metalloprotease 12 gene polymorphism and knee osteoarthritis. Unfortunately, the original MAX3 cannot account for any covariate effects. That is because it is based on the trend test and only uses information from a 2 by 3 phenotype–genotype contingency table. In the practical application of GWASs, adjusting covariates is important because it can address the issue of the confounding effect. The most straightforward way to adjust for the covariate effect is through the logistic regression. However, as the MAX3 test is not derived from the logistic regression, it remains unclear how to extend the MAX3 test to account for the covariate effect. 

The goal of this paper was to fill this research gap. Specifically, we first derived the score test based on logistic regression, which is optimal when the true genetic model is one of the three aforementioned genetic models and can be pre-determined. Then, we extended the original MAX3 test and constructed the new CMAX3 (covariate-adjusted MAX3) test based on the newly developed score test through logistic regression, which is robust against genetic model uncertainty and adjusts for the covariate effects at the same time. Moreover, the asymptotic formulas to calculate the *p*-value of the robust tests together with a user-friendly software are also released in this paper to facilitate the implementation of the proposed test in practice. A simulation study demonstrated that the proposed CMAX3 test could control the type I error rate under the null hypothesis and yet yield desirable power under the alternative hypothesis across all the commonly used genetic models (REC, ADD, and DOM). The proposed CMAX3 test was also applied to a real GWAS dataset for illustrative purposes. 

Our research was motivated by a GWAS association study from the Collaborative Study on the Genetics of Alcoholism COGA [22]. The goal of this study was to identify risk genes associated with alcohol dependence. As a complex disease, the underlying genetic model of alcohol dependence was unclear although the ADD model was used in the original analysis. Although the robust test could be used to increase the detection power, the original MAX3 test could not be applied directly to this dataset, because it contains other covariates in addition to the genotype information. This secondary data analysis requirement motivated us to develop the new statistical test proposed in this paper. 

The rest of this paper is organized as follows. We develop the new CMAX3 and its *p*-value formula in Section 2 (Method). In Section 3, we carry out simulation studies to investigate the operating characteristics of the proposed test. In Section 4, we apply the CMAX3 test to analyze a GWAS dataset. In Section 5, we demonstrate the R function to implement the proposed test in real data application. We provide a brief discussion and concluding remarks in Section 6.

## 2. Method

We consider a diallelic marker *G* and use *G* = 0, 1, 2 to denote the three genotypes aa, Aa, and AA, with A indicating the minor allele conferring high risk of the disease. Let D denote the disease status with *D* = 0 (*D* = 1) representing an unaffected (affected) individual and let *X* = ${\left(X_{1},\;\ldots X_{L}\right)}^{\prime }$ denote the vector of covariates that need to be adjusted in the model with $X_{1}\equiv 1$. We define $f_{jX}=\mathrm{Pr}(D=1|G=j,\;X)$ as the penetrance conditional on *G* = *j* and *X*, by which the recessive (REC), additive (ADD), and dominate (DOM) genetic models correspond to $f_{1X}=f_{0X},\;f_{1X}=\left(f_{0X}+f_{2X}\right)/2$, and $f_{1X}=f_{2X}$ for any value of *X*. 

When the genetic model is specified, the association between G and D can be formulated by the following logistic regression model:

(1) $$\log\left(\frac{\mathrm{Pr}\left(D=1|G,X\right)}{\mathrm{Pr}\left(D=0|G,\;X\right)}\right)=\alpha^{\prime }X+z\beta I\left(G=1\right)+\beta I\left(G=2\right)$$

where *I*() is the indicator function, $\alpha ={\left(\alpha_{1},\ldots \alpha_{L}\right)}^{\prime }$ is the covariate effect, and *β* is the genetic effect. Our objective is to test the null hypothesis $H_{0}:\;\beta =0$. 

Let $D_{i}$, $G_{i}$, and $X_{i}={\left(X_{i1},\ldots X_{iL}\right)}^{\prime }$ be the observation for patient *i* in the case-control study with a total sample size of n. We can derive the score function as

$$U\left(z\right)=\overset{n}{\underset{i=1}{\sum }}\left\{\left(zI\left(G_{i}=1\right)+I\left(G_{i}=2\right)\right)\left(I\left(D_{i}=1\right)-\hat{f_{0X_{i}}}\right)\right\}.$$

where $\hat{f_{0X_{i}}}=\frac{1}{1+\exp\left(-{\hat{\alpha }}^{\prime }X_{i}\right)}$ is the estimate of the penetrance $f_{0X_{i}}$ based on the case-control study. Furthermore, the information matrix based on model (1) can be written as:

$$I\left(z\right)=\left(\begin{array}{cc} I_{\beta }\left(z\right) & I_{\beta \alpha }{\left(z\right)}^{\prime }\\ I_{\beta \alpha }\left(z\right) & I_{\alpha } \end{array}\right)$$

$$I_{\beta }\left(z\right)=\overset{n}{\underset{i=1}{\sum }}{\left\{zI\left(G_{i}=1\right)+I\left(G_{i}=2\right)\right\}}^{2}\left(1-\hat{f_{0X_{i}}}\right)\hat{f_{0X_{i}}}$$

$$\begin{array}{cc} I_{\beta \alpha }\left(z\right)=(\overset{n}{\underset{i=1}{\sum }} & X_{i1}\{zI(G_{i}=1)+I(G_{i}\\ & =2)\left(1-\hat{f_{0X_{i}}}\right)\hat{f_{0X_{i}}},\ldots ,\;\overset{n}{\underset{i=1}{\sum }}X_{iL}\{zI\left(G_{i}=1\right)+I(G_{i}\\ & =2)\}\left(1-\hat{f_{0X_{i}}}\right)\hat{f_{0X_{i}}}{)}^{\prime } \end{array}$$

$$I_{\alpha }=\left(\begin{array}{ccc} \overset{n}{\underset{i=1}{\sum }}X_{i1}^{2}\left(1-\hat{f_{0X_{i}}}\right)\hat{f_{0X_{i}}} & \cdots & \overset{n}{\underset{i=1}{\sum }}X_{i1}X_{iL}\left(1-\hat{f_{0X_{i}}}\right)\hat{f_{0X_{i}}}\\ \vdots & \ddots & \vdots \\ \overset{n}{\underset{i=1}{\sum }}X_{i1}X_{iL}\left(1-\hat{f_{0X_{i}}}\right)\hat{f_{0X_{i}}} & \cdots & \overset{n}{\underset{i=1}{\sum }}X_{iL}^{2}\left(1-\hat{f_{0X_{i}}}\right)\hat{f_{0X_{i}}} \end{array}\right).$$

Based on the information matrix, we can derive the variance estimate of U(z) as

$$V\left(x\right)=I_{\beta }\left(z\right)-I_{\beta \alpha }{\left(z\right)}^{\prime }I_{\alpha }^{-1}I_{\beta \alpha }\left(z\right).$$

Finally, we can construct the score test as $S\left(z\right)=\frac{U\left(Z\right)}{\sqrt{V\left(z\right)}}$, which follows a standard normal distribution under the null hypothesis. Notice that *z* in the score test *S*(*z*) is a real number between 0 and 1, which represents the underlying genetic model. Specifically, we have *z* = 0, ½, and 1 as the optimal choice for the REC, ADD, and DOM model to maximize power. If the genetic model is known a priori, then we can plug in *S*(*z*) with the optimal choice of z. Unfortunately, the genetic model is often unknown for many complex diseases, and it is well known that a misclassification of the genetic model can result in a substantial power loss. To overcome this obstacle, a conventional method with two dummy variables to represent the three genotypes uses the following logistic regression model:

(2) $$\log\left(\frac{\mathrm{Pr}\left(D=1|G,X\right)}{\mathrm{Pr}\left(D=0|G,\;X\right)}\right)=\alpha^{\prime }X+\beta_{1}I\left(G=1\right)+\left(\beta_{1}+\beta_{2}\right)I\left(G=2\right)$$

Then, a likelihood ratio test (LRT) can be carried out based on model (2) to test the null hypothesis of $H_{0}:\;\beta_{1}=\beta_{2}=0$. Under the null hypothesis of no genetic effect, the LRT asymptotically follows a chi-square distribution with 2 degrees of freedom. This LRT is applicable to any underlying genetic model and, therefore, is robust. However, it is not efficient, because it ignores the natural penetrance order constraint $f_{0x}\leq f_{1x}\leq f_{2x}$, which is true for all the three genetic models (REC, ADD, and DOM).

We hereby propose a robust and efficient test referred to as the CMAX3. Let *S*(*z*) be the score test based on model (1) with pre-specified value *z*. Hence, *S*(0), *S*(1/2), and *S*(1) are the score tests optimal for the REC, ADD, and DOM models, respectively. The test statistic for CMAX3 is expressed as:

$$\mathrm{CMAX}3=\max\left(\left|S\left(0\right)\right|,\;\left|S\left(\frac{1}{2}\right)\right|,\left|S\left(1\right)\right|\right).$$

Compared with the score test *S*(*z*) based on a single genetic model, CMAX3 is robust because it considers three genetic models simultaneously and selects the best one based on the data. Compared with the LRT, CMAX3 is more efficient because it restricts the alternative space to the three commonly used genetic models (REC, ADD, and DOM) and therefore utilizes the natural penetrance order. 

Unlike *S*(*z*), which follows a standard normal distribution, or the LRT, which follows a chi-square distribution under the null hypothesis, CMAX3 does not follow any regular distribution, due to the multiple comparison and the correlations between the score tests. Hence, before using CMAX3, its asymptotic distribution under the null hypothesis must be determined first. 

Let N(0,Σ) be the asymptotic distribution of ${\left(S\left(0\right),S\left(1/2\right),\;S\left(1\right)\right)}^{\prime }$ with

$$\Sigma =\left(\begin{array}{c} \begin{array}{cc} 1 & \begin{array}{cc} \rho_{0,1/2} & \rho_{0,1} \end{array}\\ \rho_{0,1/2} & \begin{array}{cc} 1 & \rho_{1/2,1} \end{array} \end{array}\\ \begin{array}{cc} \rho_{0,1} & \begin{array}{cc} \rho_{1/2,1} & 1 \end{array} \end{array} \end{array}\right)$$

where $\rho_{z_{1},z_{2}}$ is the correlation between $S\left(z_{1}\right)$ and $S\left(z_{2}\right)$. To derive the expression of $\rho_{x_{1},x_{2}}$, we construct the following logistic regression model:

(3) $$\log\left(\frac{\mathrm{Pr}\left(D=1|G,X\right)}{\mathrm{Pr}\left(D=0|G,\;X\right)}\right)=\alpha^{\prime }X+(z_{1}\beta_{1}+z_{2}\beta_{2})I\left(G=1\right)+\left(\beta_{1}+\beta_{2}\right)I\left(G=2\right)$$

The score function based on model (3) can be expressed as

$$\begin{array}{cc} \left(\overset{n}{\underset{i=1}{\sum }}\{(z_{1}I(G_{i}=1\right) & +I\left(G_{i}=2\right))\left(I\left(D_{i}=1\right)-\hat{f_{0X_{i}}}\right)\},\overset{n}{\underset{i=1}{\sum }}\left\{\left(z_{2}I\left(G_{i}=1\right)+I\left(G_{i}=2\right)\right)\left(I\left(D_{i}=1\right)-\hat{f_{0X_{i}}}\right)\right\})\\ & =\left(U\left(z_{1}\right),U\left(z_{2}\right)\right). \end{array}$$

Therefore, $\rho_{z_{1},z_{2}}$ is the correlation coefficient between $s\left(z_{1}\right)$ and $s\left(z_{2}\right)$, which can be derived from the information matrix based on model (3). Specifically, after some calculation, the information matrix can be expressed in the form of a block matrix as follows:

$$I\left(z_{1},z_{2}\right)=\left(\begin{array}{cc} I_{\beta_{1}\beta_{2}}\left(z_{1},z_{2}\right) & I_{\beta_{1}\beta_{2}\alpha }{\left(z_{1},z_{2}\right)}^{\prime }\\ I_{\beta_{1}\beta_{2}\alpha }\left(z_{1},z_{2}\right) & I_{\alpha } \end{array}\right)$$

$$\begin{array}{c} I_{\beta_{1}\beta_{2}}\left(z_{1},z_{2}\right)\\ =\left(\begin{array}{cc} \overset{n}{\underset{i=1}{\sum }}{\left\{z_{1}I\left(G_{i}=1\right)+I\left(G_{i}=2\right)\right\}}^{2}\left(1-\hat{f_{0X_{i}}}\right)\hat{f_{0X_{i}}} & \overset{n}{\underset{i=1}{\sum }}\left\{z_{1}I\left(G_{i}=1\right)+I\left(G_{i}=2\right)\right\}\left\{z_{2}I\left(G_{i}=1\right)+I\left(G_{i}=2\right)\right\}\left(1-\hat{f_{0X_{i}}}\right)\hat{f_{0X_{i}}}\\ \overset{n}{\underset{i=1}{\sum }}\left\{z_{1}I\left(G_{i}=1\right)+I\left(G_{i}=2\right)\right\}\left\{z_{2}I\left(G_{i}=1\right)+I\left(G_{i}=2\right)\right\}\left(1-\hat{f_{0X_{i}}}\right)\hat{f_{0X_{i}}} & \overset{n}{\underset{i=1}{\sum }}{\left\{z_{2}I\left(G_{i}=1\right)+I\left(G_{i}=2\right)\right\}}^{2}\left(1-\hat{f_{0X_{i}}}\right)\hat{f_{0X_{i}}} \end{array}\right) \end{array}$$

$$I_{\beta_{1}\beta_{2}\alpha }\left(z_{1},z_{2}\right)=\left(\begin{array}{cc} \overset{n}{\underset{i=1}{\sum }}X_{i1}\left\{z_{1}I\left(G_{i}=1\right)+I\left(G_{i}=2\right)\right\}\left(1-\hat{f_{0X_{i}}}\right)\hat{f_{0X_{i}}} & \overset{n}{\underset{i=1}{\sum }}X_{iL}\left\{z_{1}I\left(G_{i}=1\right)+I\left(G_{i}=2\right)\right\}\left(1-\hat{f_{0X_{i}}}\right)\hat{f_{0X_{i}}}\\ \overset{n}{\underset{i=1}{\sum }}X_{i1}\left\{z_{2}I\left(G_{i}=1\right)+I\left(G_{i}=2\right)\right\}\left(1-\hat{f_{0X_{i}}}\right)\hat{f_{0X_{i}}} & \overset{n}{\underset{i=1}{\sum }}X_{iL}\left\{z_{2}I\left(G_{i}=1\right)+I\left(G_{i}=2\right)\right\}\left(1-\hat{f_{0X_{i}}}\right)\hat{f_{0X_{i}}} \end{array}\right).$$

By inverting the information matrix, we obtain the variance–covariance matrix of $\left(U\left(z_{1}\right),U\left(z_{2}\right)\right)$ as:

$$C\left(z_{1},z_{2}\right)=I_{\beta_{1}\beta_{2}}\left(z_{1},z_{2}\right)-I_{\beta_{1}\beta_{2}\alpha }{\left(z_{1},z_{2}\right)}^{\prime }I_{\alpha }^{-1}I_{\beta_{1}\beta_{2}\alpha }\left(z_{1},z_{2}\right).$$

Hence, the correlation coefficient $\rho_{z_{1},z_{2}}$ can be derived as

$$\rho_{z_{1},z_{2}}=\frac{\left(1,0\right)C\left(z_{1},z_{2}\right){\left(0,1\right)}^{\prime }}{\sqrt{\left(1,0\right)C\left(z_{1},z_{2}\right){\left(1,0\right)}^{\prime }\left(0,1\right)C\left(z_{1},z_{2}\right){\left(0,1\right)}^{\prime }}}.$$

Finally, with the expression of $\rho_{z_{1},z_{2}}$ at hand, we can analytically derive the *p*-value for CMAX3. In particular, let f(y,0,Σ) be the density function of the multivariate normal distribution N(0, Σ); for any observed value t>0, the *p*-value of CMAX3 can be derived as:

$$\mathrm{Pr}\left(\mathrm{CMAX}3>t|H_{0}\right)=1-\int_{-t}^{t}\int_{-t}^{t}\int_{-t}^{t}f\left(y,0,\Sigma \right)dy.$$

## 3. Simulation Studies

We conducted comprehensive simulation studies to investigate the operating characteristics of the proposed CMAX3 test. We first studied the empirical type I error rates of the proposed robust test CMAX3. We generated two covariates $X_{2}$ and $X_{3}$ following model (1). $X_{2}$ follows a binomial distribution B(0.5) and $X_{3}$ follows a uniform distribution *U*(0,1). Hence, $X_{2}$ represents a discrete variable such as gender and $X_{3}$ represents a continuous variable such as standardized age. We specified $\alpha_{1}=-5$ and β=0.

Table 1 summarizes the results. We considered four nominal significance levels such as 5 × $10^{-2}$, 1 × $10^{-2}$, 1 × $10^{-5}$, and 5 × $10^{-8}$. Within each nominal level, we varied the sample size from 2000 to 500 and the MAF from 0.1 to 0.3. We also investigated mild ($\alpha_{2}=0.25,\;\alpha_{3}=0.25$), moderate ($\alpha_{2}=1,\;\alpha_{3}=0.5$), and substantial ($\alpha_{2}=2,\;\alpha_{3}=1.5$) covariate effects. The empirical type I error rates were calculated based on $5\times 10^{9}$ replicates. As expected, the proposed CMAX3 test can consistently control type I error rates around the significance levels, which ensures the validity of the proposed tests.

Table 2 reports the empirical type I error rates of the proposed CMAX3 test and the original MAX3 test based on the trend test (denoted as OMAX3). The nominal level is fixed at 5% and the other simulation settings are the same as those used in Table 1. Based on Table 2, OMAX3 cannot control the type I error under a reasonable value and the inflation can be substantial with a significant covariate effect and/or a large sample size. Hence, in the existence of the covariate effect, the CMAX3 should be used to replace OMAX3. 

Table 3 presents the empirical power for the proposed tests in a case-control study under different genetic models (REC, ADD, and DOM). We fixed the sample size at 2000 and investigated different configurations of the covariate effect ($\alpha_{2},\;\alpha_{3}$) and the MAF. We compared the proposed robust test with the score test *S*(0), *S*(1/2), *S*(1), and the LRT. The nominal significance level was fixed at 5% and the empirical power was evaluated across 10,000 replicates. Table 3 clearly demonstrates the benefit of using CMAX3. Although the score test with a correctly specified score could always achieve the maximal power under each configuration, its power loss is also substantial when the genetic model is mis-specified, especially for the REC model. For example, when $\alpha_{2}=0.25,\;\alpha_{3}$=0.25, and MAF=0.1, although *S*(0) obtains the highest power of 81.3% when the genetic model is REC, the value plummets to 8.7% when the underling genetic model is DOM. On the other hand, *S*(1) reports the highest power of 78.1% under the DOM model but only yields a power of 14.5% under the REC model. Compared with *S*(0) and *S*(1), *S*(1/2) is less affected by the underlying genetic model. However, still, under the REC model, the power loss due to *S*(1/2) can be as large as 50% (81.3% versus 33%). Compared with the score tests, the LRT and CMAX3 hold plausible power under different genetic models and CMAX3 is consistently more powerful than the LRT. Indeed, the CMAX3 is always the second-best test under each genetic model and the power difference between CMAX3 and the optimal score test is generally limited (5–10%). Considering the fact that, in practice, it is difficult to pre-specify the genetic model, the CMAX3 is more preferred than the score test and LRT. 

## 4. Real Data Application 

We applied the proposed tests to the GWAS dataset from COGA. It is a case-control dataset containing 1399 individuals. All the case samples met lifetime DSM-IV criteria for alcohol dependence and all the control samples were not affected by alcohol or drug abuse or dependence. The data are publicly available in the dbGaP database. The original statistical analysis was conducted using logistic regression for the ADD model with sex as a covariate. Based on the original analysis, 977 SNPs had *p*-values less than $10^{-3}$ but none of them met conventional criteria for genome-wide significance. 

Here, we re-analyzed the 977 SNPs using the proposed CMAX3 test. By using the HPC cluster named IU’s Big Red II supercomputer (one petaFLOPS), it only took about 1×$10^{-3}$ s for a single SNP analysis. As a secondary data analysis, we also considered sex as the only covariate to be adjusted in the logistic regression model. For comparison, we also reported the *p*-values from the score test *S*(*x*) (*x* = 0, ½, and 1) and the LRT. Table 4 summarizes the results. Due to the page limit, we have only reported the top ten SNPs with the most significant *p*-values based on the CMAX3 test. Although there are still no genome-wide significant results, we have some interesting findings. 

First, among the ten SNPs, six show the smallest *p*-values among all the tests under the ADD model, three report the smallest *p*-values under the DOM model, and the remaining one reports the smallest *p*-value under the REC model, which indicates that any genetic model may be the underlying true model. Moreover, the *p*-values for *S*(*x*) vary substantially among different choices of x. For example, for the first SNP rs17719726 in Table 3, the *p*-value is 4.9$\times 10^{-7}$ from *S*(1) and 2.0$\times 10^{-2}$ from *S*(0), indicating that *S*(*x*) is quite sensitive to the underlying genetic model. Secondly, comparing the two robust tests, CMAX3 always yields a smaller *p*-value than the LRT does, which coincides with our finding from the simulation study that CMAX3 is more powerful than the LRT. Lastly, we also notice that CMAX3 almost always yields the second smallest *p*-values among all the tests (SNP rs2322631 is the only exception, where the *p*-values are very close between *S*(1/2) and *S*(1)). Therefore, this application clearly demonstrates that CMAX3 is a much more robust test compared with the score test *S*(*x*) and always yields a more significant *p*-value than the other robust test LRT.

## 5. Software 

In addition to the theoretical calculations, we also developed an R function to implement the proposed tests in practice. Specifically, we provide here an R function named *Rtest(data)*, which can be used to easily calculate the *p*-values for CMAX3, LRT, *S*(0), *S*(1/2), and *S*(1) from a given dataset. This function only contains one set of data, which should summarize all the genotypes, phenotypes, and covariate information. Each row of the data represents a different individual. The first column of the data records the phenotype information, with 1 representing a case status and 0 a control status. The second column of the data records the genotype information, with 0, 1, and 2 representing the number of risk alleles. The remaining columns of the data record all of the covariates.

**Figure 1:**
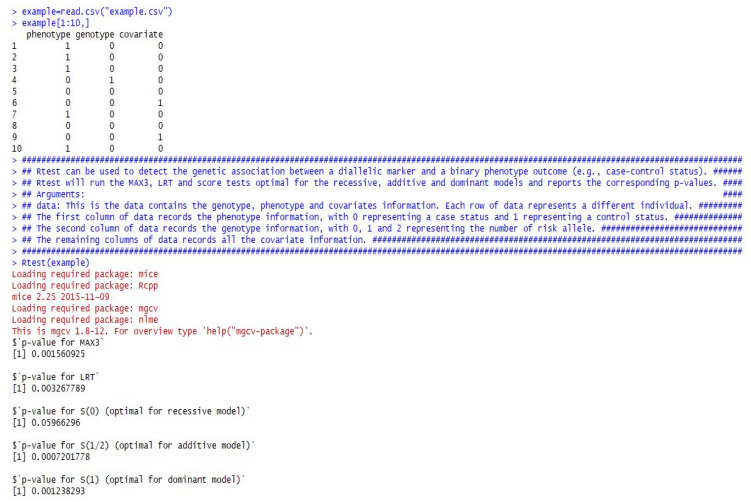
R code to analyze the example dataset.

In Figure 1, we provide a detailed example showing how to analyze the data using the provided R function. The example dataset used in Figure 1 came from the previous COGA data application. 

We should note, however, that we use 0 to represent case and 1 to represent control in the example. The R function Rtest() is indeed also applicable for a opposite notation of case-control status (e.g., 1 for case and 0 for control) because the two-sided test is used for the function and the absolute value is used to derive the *p*-value. 

## 6. Discussion and Conclusions

In this paper, we extended the original CMAX3 to adjust for covariate effects based on the likelihood function framework. The simulation results and an application to the COGA dataset demonstrated the benefit of adopting the new CMAX3 for the evaluation of genetic association in the presence of covariate effects. We also developed an *R* function to facilitate the widespread use of the proposed tests, which can be freely downloaded. The *R* code we provided can be used by researchers who can obtain the right data from the original dataset using some existing tools (e.g., PLINK). Based on the R code we provided, the researcher can also implement the proposed method in their statistical tools (e.g., *R* package) or rewrite it using other languages (e.g., *C* or *C++*). 

In this paper, we derived the distribution of the CMAX3 under the null distribution and evaluated its power using simulation studies. It is of interest to derive the asymptotic distribution under the alternative hypothesis based on which the sample size formula can be provided. In addition, we recently extended the CMAX3 to test for G-E interaction in the absence of any covariate effects [23]. It is also worth extending the proposed CMAX3 in this paper to test for G-E interaction and G-G interaction in the presence of covariate effects. 

Besides MAX3, other statistical tests are also available in the literature to tackle the genetic model uncertainty problem. For example, Wang and Sheffield [24] proposed the constrained likelihood approach (CLRT). The CLRT is a robust test that is built upon a constrained maximum likelihood in which the mean genetic effect of the heterozygous genotype is required to not exceed those of the two homozygous genotypes. In addition, Zheng and Ng [25] developed the genetic model selection approach (GMS), which is a two-phase test. In the first phase, the difference in Hardy–Weinberg disequilibrium coefficients between the cases and the controls was used for the genetic model selection. Then, an optimal trend test corresponding to the selected model was used for testing association. The correlation of the statistics used for selection and the test for association was derived to adjust the two-phase analysis with control of the Type-I error rate. Similar to the original MAX3, neither the CLRT nor GMS could account for the covariate effects, so extending CLRT and GMS is another possible direction for our future work. 

As pointed by one reviewer, So and Sham [26] proposed another robust test allowing for covariates. Although the proposed method looks similar to those of So and Sham, their two methods were developed under different statistical models. The proposed test was developed under a statistical rigorous likelihood function framework, whereas So and Sham’s test was based on an empirical Monte–Carlo approximation algorithm. Therefore, it is more straightforward to extend the proposed CMAX3 test to handle other problems (e.g., G-G and G-E interaction). Nevertheless, to the best of our knowledge, So and Sham’s test is the first work in the field of statistical genetics to construct the robust test adjusting for covariate effects, which motivates us to further investigate this problem.

## Figures and Tables

**Table 1:** Empirical type I error rates of CMAX3 with different nominal levels.

Nominal	Sample	$\alpha_{2}=0.25$	$\alpha_{3}=0.25$	$\alpha_{2}=1$	$\alpha_{3}=0.5$	$\alpha_{2}=2$	$\alpha_{3}=1.5$
level	size	MAF = 0.1	MAF = 0.3	MAF = 0.1	MAF = 0.3	MAF = 0.1	MAF = 0.3
$5\times 10^{-2}$	2000	$4.6\times 10^{-2}$	$5.2\times 10^{-2}$	$4.3\times 10^{-2}$	$5.3\times 10^{-2}$	$4.8\times 10^{-2}$	$4.9\times 10^{-2}$
	1000	$5.2\times 10^{-2}$	$5.5\times 10^{-2}$	$5.2\times 10^{-2}$	$4.9\times 10^{-2}$	$5.6\times 10^{-2}$	$5.0\times 10^{-2}$
	500	$4.3\times 10^{-2}$	$5.2\times 10^{-2}$	$5.4\times 10^{-2}$	$4.9\times 10^{-2}$	$4.7\times 10^{-2}$	$5.4\times 10^{-2}$
$1\times 10^{-2}$	2000	$1.0\times 10^{-2}$	$1.1\times 10^{-2}$	$0.8\times 10^{-2}$	$1.0\times 10^{-2}$	$1.1\times 10^{-2}$	$0.9\times 10^{-2}$
	1000	$1.2\times 10^{-2}$	$1.3\times 10^{-2}$	$1.3\times 10^{-2}$	$0.9\times 10^{-2}$	$0.7\times 10^{-2}$	$1.3\times 10^{-2}$
	500	$0.8\times 10^{-2}$	$0.7\times 10^{-2}$	$1.1\times 10^{-2}$	$1.0\times 10^{-2}$	$0.8\times 10^{-2}$	$1.1\times 10^{-2}$
$1\times 10^{-5}$	2000	$0.9\times 10^{-5}$	$1.0\times 10^{-5}$	$0.7\times 10^{-5}$	$1.2\times 10^{-5}$	$1.1\times 10^{-5}$	$1.1\times 10^{-5}$
	1000	$1.1\times 10^{-5}$	$1.2\times 10^{-5}$	$1.0\times 10^{-5}$	$1.2\times 10^{-5}$	$1.3\times 10^{-5}$	$1.1\times 10^{-5}$
	500	$0.9\times 10^{-5}$	$1.3\times 10^{-5}$	$1.0\times 10^{-5}$	$1.1\times 10^{-5}$	$0.8\times 10^{-5}$	$1.1\times 10^{-5}$
$5\times 10^{-8}$	2000	$5.2\times 10^{-8}$	$5.5\times 10^{-8}$	$4.8\times 10^{-8}$	$5.3\times 10^{-8}$	$5.5\times 10^{-8}$	$4.9\times 10^{-8}$
	1000	$5.3\times 10^{-8}$	$5.7\times 10^{-8}$	$4.3\times 10^{-8}$	$4.8\times 10^{-8}$	$5.7\times 10^{-8}$	$5.3\times 10^{-8}$
	500	$4.7\times 10^{-8}$	$4.9\times 10^{-8}$	$5.5\times 10^{-8}$	$6.1\times 10^{-8}$	$4.8\times 10^{-8}$	$5.1\times 10^{-8}$

**Table 2:** Empirical type I error rates of CMAX3 and OMAX3 with nominal levels 5%.

Test	Sample	$\alpha_{2}=0.25$	$\alpha_{3}=0.25$	$\alpha_{2}=1$	$\alpha_{3}=0.5$	$\alpha_{2}=2$	$\alpha_{3}=1.5$
	size	MAF = 0.1	MAF = 0.3	MAF = 0.1	MAF = 0.3	MAF = 0.1	MAF = 0.3
CMAX3	2000	$4.6\times 10^{-2}$	$5.2\times 10^{-2}$	$4.3\times 10^{-2}$	$5.3\times 10^{-2}$	$4.8\times 10^{-2}$	$4.9\times 10^{-2}$
	1000	$5.2\times 10^{-2}$	$5.5\times 10^{-2}$	$5.2\times 10^{-2}$	$4.9\times 10^{-2}$	$5.6\times 10^{-2}$	$5.0\times 10^{-2}$
	500	$4.3\times 10^{-2}$	$5.2\times 10^{-2}$	$5.4\times 10^{-2}$	$4.9\times 10^{-2}$	$4.7\times 10^{-2}$	$5.4\times 10^{-2}$
OMAX3	2000	$1.3\times 10^{-1}$	$1.8\times 10^{-1}$	$2.8\times 10^{-1}$	$3.3\times 10^{-1}$	$3.4\times 10^{-1}$	$4.2\times 10^{-1}$
	1000	$9.4\times 10^{-2}$	$1.5\times 10^{-1}$	$1.6\times 10^{-1}$	$2.1\times 10^{-1}$	$2.6\times 10^{-1}$	$3.3\times 10^{-1}$
	500	$6.7\times 10^{-2}$	$6.4\times 10^{-2}$	$7.8\times 10^{-2}$	$8.1\times 10^{-2}$	$9.4\times 10^{-2}$	$9.1\times 10^{-2}$

**Table 3:** Empirical powers from simulation study using sample size 2000 and nominal level 5%.

		$\alpha_{2}=0.25$	$\alpha_{3}=0.25$	$\alpha_{2}=1$	$\alpha_{3}=0.5$	$\alpha_{2}=2$	$\alpha_{3}=1.5$
Test	Model	MAF = 0.1	MAF = 0.3	MAF = 0.1	MAF = 0.3	MAF = 0.1	MAF = 0.3
*S*(0)	REC	81.3	94.7	82.4	93.3	63.5	86.6
*S*(1/2)		33.0	72.6	29.6	69.6	21.9	58.6
*S*(1)		14.5	23.6	11.7	21.9	8.0	17.8
LRT		71.4	86.8	71.8	86.8	52.1	76.5
CMAX3		75.0	90.9	75.3	89.8	55.4	80.5
*S*(0)	ADD	18.1	49.7	19.5	48.5	16.4	48.1
*S*(1/2)		70.0	81.9	71.8	82.1	59.6	77.7
*S*(1)		61.4	74.6	62.5	74.2	50.8	68.4
LRT		58.3	73.5	59.7	72.0	47.8	67.3
CMAX3		66.3	78.5	69.5	76.8	54.4	72.7
*S*(0)	DOM	8.7	13.1	7.4	11.7	8.2	11.8
*S*(1/2)		71.0	72.6	69.8	71.0	57.5	63.2
*S*(1)		78.1	81.2	78.0	77.9	66.8	73.0
LRT		67.6	70.7	66.9	69.9	55.5	62.5
CMAX3		76.9	74.2	75.6	74.2	64.6	68.2

**Table 4:** Top ten SNPs with most significant *p*-values based on the CMAX3 test. For each SNP, the smallest *p*-value obtained by all methods is in bold.

			Test		
SNPs	MAX3	LRT	*S*(0)	*S*(1/2)	*S*(1)
rs17719726	$9.6\times 10^{-7}$	$2.3\times 10^{-6}$	$2.0\times 10^{-2}$	$1.1\times 10^{-6}$	** $4.9\times 10^{-7}$ **
rs2082371	$1.4\times 10^{-6}$	$4.2\times 10^{-6}$	$6.4\times 10^{-2}$	$3.1\times 10^{-6}$	** $7.0\times 10^{-7}$ **
rs2322631	$2.1\times 10^{-6}$	$3.8\times 10^{-6}$	$5.2\times 10^{-3}$	** $1.1\times 10^{-6}$ **	$1.4\times 10^{-6}$
rs13247743	$3.5\times 10^{-6}$	$1.0\times 10^{-5}$	$1.6\times 10^{-1}$	$3.7\times 10^{-6}$	$1.7\times 10^{-6}$
rs28760505	$5.9\times 10^{-6}$	$1.5\times 10^{-5}$	$1.7\times 10^{-3}$	** $2.9\times 10^{-6}$ **	$1.2\times 10^{-5}$
rs7577225	$6.1\times 10^{-6}$	$1.4\times 10^{-5}$	$2.9\times 10^{-3}$	** $3.2\times 10^{-6}$ **	$7.6\times 10^{-6}$
rs1195812	$9.7\times 10^{-6}$	$2.8\times 10^{-5}$	$1.4\times 10^{-4}$	** $5.0\times 10^{-6}$ **	$2.4\times 10^{-4}$
rs12561944	$1.3\times 10^{-5}$	$2.5\times 10^{-5}$	** $6.5\times 10^{-6}$ **	$8.6\times 10^{-4}$	$3.5\times 10^{-2}$
rs7698703	$1.5\times 10^{-5}$	$4.4\times 10^{-5}$	$8.1\times 10^{-4}$	** $7.5\times 10^{-6}$ **	$9.8\times 10^{-5}$
rs701837	$1.6\times 10^{-5}$	$4.6\times 10^{-5}$	$7.8\times 10^{-4}$	** $8.0\times 10^{-6}$ **	$1.1\times 10^{-4}$

## Data Availability

The real data are publicly available in the dbGaP database from https://www.ncbi.nlm.nih.gov/gap/?term=COGA (accessed on 25 October 2021). The R function to facilitate the widespread use of the proposed tests can be freely downloaded at https://github.com/yongzang2020/MAX3_covariates (accessed on 25 October 2021).
